# Changes in proteinuria and the associated risks of ischemic heart disease, acute myocardial infarction, and angina pectoris in Korean population

**DOI:** 10.4178/epih.e2023088

**Published:** 2023-09-30

**Authors:** Sung Keun Park, Ju Young Jung, Min-Ho Kim, Chang-Mo Oh, Eunhee Ha, Eun Hye Yang, Hyo Choon Lee, Soonsu Shin, Woo Yeon Hwang, Sangho Lee, So Youn Shin, Jae-Hong Ryoo

**Affiliations:** 1 Total Healthcare Center, Kangbuk Samsung Hospital, Sungkyunkwan University School of Medicine, Seoul, Korea; 2 Department of Preventive Medicine, Graduate School, Kyung Hee University, Seoul, Korea; 3 Informatization Department, Ewha Womans University Seoul Hospital, Seoul, Korea; 4 Department of Preventive Medicine, Kyung Hee University School of Medicine, Seoul, Korea; 5 Department of Occupational and Environment Medicine, Ewha Womans University College of Medicine, Seoul, Korea; 6 Department of Occupational and Environmental Medicine, Kyung Hee University Hospital, Seoul, Korea; 7 Department of Obstetrics and Gynecology, Kyung Hee University Hospital, Seoul, Korea; 8 Department of Anesthesiology and Pain Medicine, Kyung Hee University Medical Center, Seoul, Korea; 9 Department of Radiology, Kyung Hee University School of Medicine, Seoul, Korea; 10 Department of Occupational and Environmental Medicine, Kyung Hee University School of Medicine, Seoul, Korea

**Keywords:** Angina pectoris, Myocardial infarction, Myocardial ischemia, Proteinuria

## Abstract

**OBJECTIVES:**

Proteinuria is widely used to predict cardiovascular risk. However, there is insufficient evidence to predict how changes in proteinuria may affect the incidence of cardiovascular disease.

**METHODS:**

The study included 265,236 Korean adults who underwent health checkups in 2003-2004 and 2007-2008. They were categorized into 4 groups based on changes in proteinuria (negative: negative → negative; resolved: proteinuria ≥1+ → negative; incident: negative → proteinuria ≥1+; persistent: proteinuria ≥1+ → proteinuria ≥1+). We conducted 6 years of follow-up to identify the risks of developing ischemic heart disease (IHD), acute myocardial infarction (AMI), and angina pectoris according to changes in proteinuria. A multivariate Cox proportional-hazards model was used to calculate adjusted hazard ratios (HRs) and 95% confidence intervals (CIs) for incident IHD, AMI, and angina pectoris.

**RESULTS:**

The IHD risk (expressed as HR [95% CI]) was the highest for persistent proteinuria, followed in descending order by incident and resolved proteinuria, compared with negative proteinuria (negative: reference, resolved: 1.211 [95% CI, 1.104 to 1.329], incident: 1.288 [95% CI, 1.184 to 1.400], and persistent: 1.578 [95% CI, 1.324 to 1.881]). The same pattern was associated with AMI (negative: reference, resolved: 1.401 [95% CI, 1.048 to 1.872], incident: 1.606 [95% CI, 1.268 to 2.035], and persistent: 2.069 [95% CI, 1.281 to 3.342]) and angina pectoris (negative: reference, resolved: 1.184 [95% CI, 1.065 to 1.316], incident: 1.275 [95% CI, 1.160 to 1.401], and persistent: 1.554 [95% CI, 1.272 to 1.899]).

**CONCLUSIONS:**

Experiencing proteinuria increased the risks of IHD, AMI, and angina pectoris even after proteinuria resolved.

## GRAPHICAL ABSTRACT

[Fig f1-epih-45-e2023088]

## INTRODUCTION

Ischemic heart disease (IHD) refers to a reduction in blood flow to the heart muscle, primarily due to atherosclerotic changes in the coronary artery [1]. IHD, also called coronary artery disease, encompasses a broad spectrum of clinical conditions, including angina pectoris and acute myocardial infarction [2]. IHD is a leading cause of death worldwide, accounting for a substantial proportion of cardiovascular (CV) mortality [3]. Therefore, identifying modifiable and non-modifiable risk factors for IHD is clinically important to mitigate burdens related to this disease.

Proteinuria is a surrogate marker for chronic kidney disease (CKD) and predicts the progression of end-stage renal disease [4,5]. It is widely accepted that proteinuria is an independent risk factor for CV morbidity and mortality in patients [6]. Moreover, evidence from the general population suggests that proteinuria is associated with a higher risk of all-cause mortality and adverse CV outcomes, even in individuals without CKD [7,8]. A meta-analysis showed that a urine dipstick proteinuria result of 1+ or more was associated with a 50% increase in the risk of coronary heart disease after adjusting for conventional risk factors in 169,949 individuals [9]. Those studies analyzed the CV risk based on the amount of proteinuria assessed at a specific time. However, proteinuria is not static and can vary over time. The degree of proteinuria may be affected by antihypertensive medication, treatment of renal disease, smoking, and adiposity [10-12]. In addition, functional and physiological proteinuria can disappear spontaneously. The prognostic utility of changes in proteinuria remains a matter of debate. In particular, it is uncertain whether changes in proteinuria can predict the future development of cardiovascular disease (CVD). Although some studies have evaluated the association between changes in proteinuria and CV risk in patients with diabetes, prediabetes, hypertension, and vascular disease [13-16], data derived from the general population are still limited.

To identify the effects of changes in proteinuria on CVD, we quantified the risk of IHD according to proteinuria variation over time in 265,236 Korean adults. In the present study, the dependent variables included acute myocardial infarction (AMI) and angina pectoris, which were used to describe the relationship between changes in proteinuria and IHD subtypes.

## MATERIALS AND METHODS

### Data sources

In Korea, the National Health Insurance Service (NHIS) is operated by the National Health Insurance Corporation (NHIC), which offers the NHIS to the Korean population. NHIS covers more than 97% of Korean residents, and its medical information is stored in the National Health Information Database (NHID). Therefore, the NHID represents the medical service usage of the entire Korean population [17]. Korean adults over 40 years of age should receive an NHIS-supported health checkup annually or biennially.

By contract with the NHIC, all Korean medical institutions provide the medical information of their healthcare users and patients to the NHID. Therefore, the NHID is a public database containing medical information and socio-demographic variables for the Korean population from healthcare utilization and health checkups. In recent years, the NHIS in Korea has provided a sampled database for research purposes after deleting personally identifiable information. The sampled data used in the current study were provided by the NHIC, including information in Statistics Korea on health checkups and links to the development of IHD, AMI, and angina pectoris.

### Study participants

The study participants were Korean adults who underwent at least 2 health checkups in 2003-2004 and 2007-2008. We initially enrolled 302,237 participants whose records of urine dipstick proteinuria could be identified at each health checkup within the years 2003-2004 and 2007-2008. Of these, we excluded 37,001 participants who were previously diagnosed with IHD (I20-I25) from 2002 to a date preceding the medical health checkup in 2007-2008. Finally, 265,236 participants were included in the final analysis and monitored for incident IHD, AMI, and angina pectoris based on diagnostic codes recorded in the NHID, which uses the International Classification of Diseases (ICD), 10th revision, Clinical Modification (ICD-10-CM). The total durations of follow-up were 1,433,750.3 person-years for IHD, 1,509,883.7 person-years for AMI, and 1,451,986.6 person-years for angina pectoris, respectively. Definition of changes in proteinuria Information on proteinuria was obtained from a urine dipstick test performed during a biennial health checkup. According to the urine dipstick test at each of the health checkups in 2003-2004 and 2007-2008, study participants were categorized into 2 groups (negative and proteinuria ≥ 1+) at each health checkup. The study participants were further categorized into 4 groups based on the changes in proteinuria between the first checkup (2003-2004) and second checkup (2007-2008): negative (negative → negative), resolved (proteinuria ≥ 1+ → negative), incident (negative → proteinuria ≥ 1+), and persistent (proteinuria ≥ 1+ → proteinuria ≥ 1+).

### Health survey examinations and laboratory measurements

Baseline demographic, anthropometric, and laboratory data were obtained from the 2007-2008 health checkups. The checkups included a questionnaire on lifestyle and medical history, anthropometric measurements, and laboratory measurements. Based on the smoking-related questionnaire, participants were categorized according to their smoking status as never-smokers, ex-smokers, or current smokers. Alcohol intake was defined as consumption ≥ 3 times per week. Physical activity was defined as engaging in moderate-intensity physical activity for at least 30 minutes per day for at least 4 days each week.

Antiplatelet medications were deemed relevant when prescriptions were received for longer than 30 days and consisted of aspirin, clopidogrel, ticagrelor, cilostazol, or prasugrel. Anticoagulant medications were considered relevant when prescriptions were received for > 30 days and consisted of warfarin, apixaban, rivaroxaban, or endoxaban. Body mass index (BMI) was calculated as the weight (kg) divided by the height squared (m2). Trained examiners measured systolic blood pressure (BP) and diastolic BP. The following laboratory data were measured while the participants underwent health examinations: fasting blood glucose, total cholesterol, aspartate aminotransferase (AST), alanine aminotransferase (ALT), and γ-glutamyltransferase (GGT).

### Outcome definitions

The NHID data were linked to data on diagnosed diseases from Statistics Korea. In this study, the date of study entry was the date of the last health checkup in 2007-2008 (the baseline). Follow-up was conducted up to the date of identifying incident IHD, AMI, and angina pectoris in participants with those issues, and up to December 31, 2013, in participants without incident IHD, AMI, or angina pectoris.

Identification of incident IHD, AMI, and angina pectoris was based on ICD-10-CM, registered in the NHID as follows: IHD (I20-I25), AMI (I21-I22), and angina pectoris (I20). IHD, AMI, and angina pectoris were diagnosed by physicians, and ICD-10 codes were assigned simultaneously.

### Statistical analysis

Data are expressed as the mean± standard deviation or median (interquartile range) for continuous variables and as percentages of the number for categorical variables. One-way analysis of variance and the chi-square test were used to analyze the statistical significance of differences among participants’ characteristics at the time of enrollment in relation to the 4 groups of changes in proteinuria. The person-years were calculated as the sum of follow-up times from the baseline until a new diagnosis of IHD, AMI, or angina pectoris, or until December 31, 2013.

To evaluate the associations among changes in proteinuria and the risks of incident IHD, AMI, and angina pectoris, we used Cox proportional-hazards models to estimate adjusted hazard ratios (HRs) and 95% confidence intervals (CIs) for incident IHD, AMI, and angina pectoris. These models were adjusted for multiple confounding factors. In multivariate models, we included variables that might confound the relationship between changes in proteinuria and incident IHD, AMI, and angina pectoris, including age, sex, BMI, systolic BP, fasting blood glucose, total cholesterol, GGT, smoking status, alcohol intake, physical activity, antiplatelet medications, and anticoagulant medications.

To minimize potential reverse causality, we conducted an additional analysis excluding participants who developed IHD, AMI, or angina pectoris within 1 year of cohort entry (n= 5,644 in IHD, n= 438 in AMI, and n= 4,225 in angina pectoris). The reanalyzed data are presented in the Supplementary Materials 1-3.

To test the validity of the Cox proportional hazards model, we checked the proportional-hazards assumption. It was assessed using the log-minus-log survival function and found to be graphically unviolated. Statistical significance was set at p< 0.05. All statistical analyses were performed using SAS version 9.4 (SAS Institute Inc., Cary, NC, USA).

### Ethics statement

Ethics approvals for the study protocol and data analysis were obtained from the Institutional Review Board of Kyung Hee University Hospital. The institutional review board waived the requirement for informed consent because researchers accessed a de-identified database retrospectively for the study.

## RESULTS

During follow-up between 2007-2008 and 2013, there were 26,547 cases of incident IHD (10.01%), 2,356 cases of incident AMI (0.89%), and 20,912 cases of incident angina pectoris (7.88%). Table 1 presents the baseline characteristics of the study participants in relation to the changes in proteinuria in the 4 groups. The study participants were characterized by middle age (mean age of 56.6±8.7 years), and the majority of participants were males (56.4%). Compared with the negative proteinuria group, the other groups were older and had higher values for BMI, BP (systolic and diastolic), total cholesterol, fasting blood glucose, and GGT. The incidence of IHD, AMI, and angina pectoris was highest in those with persistent proteinuria, followed in descending order by those with incident, resolved, and negative proteinuria.

Table 2 shows the HRs and 95% CIs for the incidence of IHD according to the 4 groups classified by changes in proteinuria. In the fully adjusted analysis, the risk of IHD was highest for those with persistent proteinuria, followed in descending order by those with incident and resolved proteinuria, compared with negative proteinuria (reference) (resolved: 1.211 [95% CI, 1.104 to 1.329], incident: 1.288 [95% CI, 1.184 to 1.400], and persistent: 1.578 [95% CI, 1.324 to 1.881], p for trend <0.001). This association was stronger between changes in proteinuria and incident AMI (Table 3). Compared with negative proteinuria, the other groups were significantly associated with a higher risk of AMI (negative: reference, resolved: 1.401 [95% CI, 1.048 to 1.872], incident: 1.606 [95% CI, 1.268 to 2.035], and persistent: 2.069 [95% CI, 1.281 to 3.342], p for trend < 0.001). The risk of angina pectoris was also highest in the persistent group, followed by the incident and resolved proteinuria groups (negative: reference, resolved: 1.184 [95% CI, 1.065 to 1.316], incident: 1.275 [95% CI, 1.160 to 1.401], and persistent: 1.554 [95% CI, 1.272 to 1.899], p for trend < 0.001; Table 4).

The Supplementary Materials 1-3 show the results of the additional analysis excluding participants who were diagnosed with IHD, AMI, or angina pectoris within 1 year after the second urine dipstick test (time of cohort entry) to minimize potential reverse causality. Consistent results were found; specifically, compared with negative proteinuria, the risks of IHD (Supplementary Material 1), AMI (Supplementary Material 2), and angina pectoris (Supplementary Material 3) were highest in those with persistent proteinuria, followed in descending order by those with incident and resolved proteinuria.

## DISCUSSION

Through the follow-up of 265,236 Korean adults across 6 years, we performed a longitudinal analysis of the risks of developing IHD, AMI, and angina pectoris according to changes in proteinuria assessed by urine dipstick across 4 years. Our analysis indicated that all patterns of changes in proteinuria were associated significantly with a higher risk of developing IHD, AMI, and angina pectoris, even after adjusting for confounding factors such as age, sex, BMI, systolic BP, fasting blood glucose, total cholesterol, GGT, smoking status, alcohol intake, physical activity, antiplatelet medications, and anticoagulant medications. Among changes in proteinuria, persistent proteinuria (proteinuria ≥ 1+ → proteinuria ≥ 1+) showed the highest risk, more than 70%, followed by incident proteinuria (negative → proteinuria ≥ 1+), with more than a 30% risk elevation. This result suggests that new-onset proteinuria and persistent proteinuria are associated with a higher risk of developing IHD, AMI, and angina pectoris.

Previous studies have reported consistent results. The presence of proteinuria was reported to be a predictor of CVD across ethnicities [18,19], which supports the association between new-onset proteinuria and a higher risk of IHD. In a prospective followup of 60 months in Japanese adults under 75 years of age, moderately higher albuminuria assessed by the urine albumin-to-creatinine ratio (UACR) was an independent predictor of CV events, including stroke, myocardial infarction (MI), revascularization, and CV death [18]. The risk of adverse CV outcomes, including MI, was independently associated with higher levels of urine dipstick proteinuria at a given level of kidney function in a community-based cohort study of 920,985 Canadian adults [19]. Moreover, several studies have shown that new-onset proteinuria and persistent proteinuria were strong risk factors for CV mortality and morbidity. Wang et al. [13] investigated the association between changes in urine dipstick proteinuria over 2 years and MI, in which persistent proteinuria was significantly associated with a higher risk of MI among 17,625 individuals with prediabetes or diabetes. In 23,480 patients with vascular disease or high-risk diabetes, a 2-fold or greater increase in albuminuria assessed by UACR was associated with an approximately 50% higher rate of mortality and CV events [14]. In urine dipstick testing, persistent 1+ proteinuria and incident 1+ proteinuria were associated with 3.49-fold (1.64-7.41) greater and 1.87-fold (0.92-3.78) greater mortality over 9-10 years, respectively [20]. Although a urine dipstick test is inferior to UACR and 24-hour urine collection in quantifying urinary albumin excretion precisely, it is evident that a urine dipstick test reflects proteinuria, similar to measurements that quantify urinary albumin excretion. Therefore, the aforementioned studies suggest that incident proteinuria and persistent proteinuria are potential predictors of CV events. Nonetheless, results from the general population are scarce, and published data are insufficient to identify the effects of changes in proteinuria on the risk of IHD. Our results were derived from the general Korean population with a large sample size, which may expand the clinical significance of incident and persistent proteinuria to the general population.

Several mechanisms may explain the role of persistent proteinuria and new-onset proteinuria in predicting IHD. Proteinuria is regarded as a surrogate marker reflecting systemic endothelial dysfunction and atherosclerotic changes that are responsible for the pathophysiology of IHD. It has been hypothesized that urinary protein excretion reflects not only localized subclinical renal disease but also more generalized vascular endothelial dysfunction [21,22]. Proteinuria has been linked to inflammatory biomarkers that can cause endothelial dysfunction by inhibiting nitric oxide production [23,24]. Additionally, thrombogenic factors have been implicated as potential mechanisms underlying the relationship between proteinuria and IHD. Activation of thrombotic molecules contributes to atherosclerotic changes in coronary arteries [25]. Studies have suggested that proteinuria is linked to the activation of endothelial prothrombotic molecules, platelet adhesiveness, and erythrocyte aggregation, which are postulated to increase the risk of thrombosis in the coronary artery [23,26,27]. In addition to endothelial dysfunction and atherosclerosis, proteinuria is associated with insulin resistance, which can contribute to the pathogenesis of IHD [28,29]. Therefore, it is inferred that persistent proteinuria and newly developed proteinuria reflect vascular endothelial dysfunction, atherosclerosis, and insulin resistance, thereby predicting higher risks of developing IHD, AMI, and angina pectoris. In particular, persistent proteinuria may have a longer-lasting effect on the coronary arteries than new-onset proteinuria and resolved proteinuria, which results in the highest risk.

In contrast to the effects of incident and persistent proteinuria, it is unclear whether the remission of proteinuria (reduced or resolved proteinuria) leads to a lower risk of IHD. Some studies have reported the potential benefits of proteinuria remission for improving CV risk. A meta-regression analysis of 32 randomized trials showed that a 10% reduction in urinary albumin excretion assessed by UACR and 24-hour urine collection was associated with a 13% decrease in MI incidence in patients with diabetes or hypertension [15]. In patients with type 2 diabetes and vascular disease, the degree of albuminuria reduction assessed by UACR and 24-hour collection was correlated with a lower incidence of CV events [14,16]. However, conflicting results have indicated that albuminuria remission is not directly linked to an improvement in CV prognosis. *Post hoc* analyses in patients with diabetes showed that remission of albuminuria assessed by UACR was not associated with a lower risk of CVD [30,31]. Moreover, a study reported that the risk of CVD continued to increase even after albuminuria remission [30]. A meta-analysis raised the concern that the treatment effect of reducing proteinuria might be overestimated for improving CV prognosis [32]. In studies analyzing the association between changes in proteinuria and CV risk, persistent proteinuria increased the risk of MI and CV mortality, whereas resolved proteinuria did not show a significant association with MI and CV mortality [13,20]. Our analysis showed that participants whose proteinuria disappeared (resolved proteinuria) still experienced more than a 20% greater risk of developing IHD, AMI, and angina pectoris. This finding suggests that the risk of IHD persisted even after proteinuria remission. The reduction in proteinuria is not likely to be directly linked to the alleviation of the pathophysiological process of CVD. The manifestation of proteinuria indicates the activation of the underlying mechanism of CVD, which can persist even after proteinuria resolves. Although participants with resolved proteinuria had a relatively lower risk of IHD than those with persistent proteinuria, close monitoring and management of CV risk may be needed in patients with resolved proteinuria.

The strengths of our study are accuracy in the diagnosis of diseases using ICD-10 codes and the reliability of demographic and laboratory measurements obtained during the national health checkups. We enhanced the reliability of the results by confirming identical patterns of relationships in an additional analysis (Supplementary Materials 1-3) that minimized reverse causality.

Nonetheless, several limitations should be recognized in the present study. First, the urine dipstick test was used to evaluate proteinuria. The urine dipstick test is appropriate for screening the general population owing to its convenience and cost-effectiveness. It has been reported that the urine dipstick test for proteinuria ≥ 1+ had high negative predictability and specificity for UACR ≥ 30 mg/g [33]. However, the urine dipstick test has also been reported to have relatively low sensitivity and high false positive predictability for UACR ≥ 30 mg/g [33,34], and it is less precise than UACR or 24-hour urine collection in quantifying urine protein excretion. Thus, further studies should evaluate the risk of IHD according to changes in proteinuria measured quantitatively. Second, it was impossible to consider every factor potentially affecting the risk of IHD. These factors include dietary habits, family history, anti-hypertensive medications, use of anticoagulants, and comorbidities such as coagulation disorders and other CVD. Nonetheless, we failed to adjust for these factors due to an absence of information in our raw data. Third, the follow-up period of the present study was about 6 years, which is not enough to reflect the long-term risk of incident IHD. In our study, the average incidence rates of IHD, AMI, and angina pectoris were 10.01%, 0.89%, and 7.88%, respectively. A longer follow-up might have shown a higher incidence of diseases, which would have increased the statistical power of our analysis.

In conclusion, all patterns of changes in proteinuria were associated significantly with higher risks of developing IHD, AMI, and angina pectoris, even in cases of resolved proteinuria. Persistent proteinuria was associated with the highest risk, followed by incident proteinuria and resolved proteinuria. These findings suggest that participants who manifested proteinuria even once remained at an elevated risk of developing coronary artery disease, regardless of subsequent proteinuria changes.

## Figures and Tables

**Figure f1-epih-45-e2023088:**
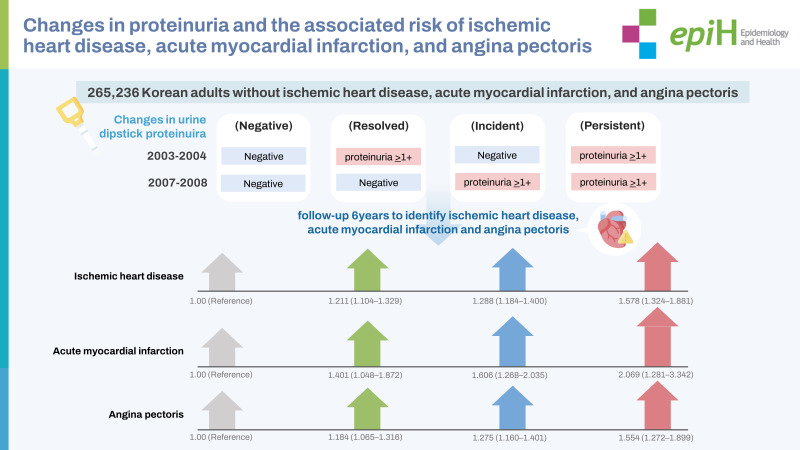


**Table 1.** Baseline clinical characteristics of study participants according to changes in proteinuria (n=265,236)

Characteristics		Overall	Categories of changes in proteinuria (from 2003-2004 to 2007-2008)^1^				
			Negative (n=256,865)	Resolved (n=3,542)	Incident (n=4,154)	Persistent (n=675)	p for trend^2^
Total PYs for IHD		1,433,750.3	1,390,097.8	18,775.2	21,551.3	3,326.0	
Average PYs for IHD		5.4±1.2	5.4 ±1.2	5.30±0.4	5.19±1.5	4.92±1.7	<0.001
Total PYs for AMI		1,509,883.7	1,462,809.1	20,160.1	23,223.1	3,691.4	
Average PYs for AMI		5.69±0.8	5.70±0.8	5.69±0.9	5.59±1.0	5.47±1.2	<0.001
Total PYs for angina pectoris		1,451,986.6	1,407,427.2	19,119.9	22,010.6	3,428.9	
Average PYs for angina pectoris		5.47±1.1	5.48±1.1	5.40±1.3	5.30±1.4	5.08±1.7	<0.001
Age (yr)		56.6±8.7	56.5±8.7	58.0±9.1	58.0±9.2	58.7±9.2	<0.001
Sex							<0.001
	Male	149,509 (56.4)	144,605 (56.3)	1,891 (53.4)	2,552 (61.4)	461 (68.3)	
	Female	115,727 (43.6)	112,260 (43.7)	1,651 (46.6)	1,602 (38.6)	214 (31.7)	
BMI (kg/m^2^)		23.9±2.9	23.9±2.9	24.5±3.1	24.4±3.2	25.0±3.4	<0.001
Systolic BP (mmHg)		125.4±15.8	125.2±15.7	128.2±16.9	130.5±17.7	134.5±18.5	<0.001
Diastolic BP (mmHg)		78.0±10.2	77.9±10.2	79.2±10.8	80.6±11.3	81.5±11.3	<0.001
Total cholesterol (mg/dL)		198.8±36.5	198.7±36.4	199.1±38.6	204.7±44.0	203.2±43.1	<0.001
Fasting blood glucose (mg/dL)		99.0±25.5	98.7±24.8	105.3±33.3	113.5±43.6	120.1±47.1	<0.001
AST (U/L)		23 (20-29)	23 (20-29)	24 (20-30)	24 (20-31)	23 (20-30)	<0.001
ALT (U/L)		21 (16-29)	21 (16-29)	21 (16-31)	22 (16-32)	23 (16-31)	<0.001
GGT (U/L)		25 (17-41)	24 (16-40)	26 (17-45)	30 (19-54)	31 (20-53)	<0.001
Smoking status							<0.001
	Never-smoker	180,510 (71.8)	174,904 (71.8)	2,486 (73.7)	2,691 (68.8)	429 (67.0)	
	Ex-smoker	24,322 (9.6)	23,520(9.6)	311 (9.2)	412 (10.5)	79 (12.3)	
	Current smoker	46,487 (18.5)	44,977 (18.4)	575 (17.0)	803 (20.5)	132 (20.6)	
Alcohol intake		26,209 (10.0)	25,313 (10.0)	347 (9.9)	484 (11.9)	65 (9.7)	0.003
Physical activity		64,533 (24.7)	62,375 (24.7)	923 (26.4)	1,072 (26.2)	163 (24.4)	0.009
Antiplatelet medications		26,252 (9.9)	24,684 (9.6)	660 (18.6)	705 (16.9)	203 (30.0)	<0.001
Anticoagulant medications		539 (0.2)	501 (0.2)	13 (0.3)	18 (0.4)	7 (1.0)	<0.001
Incident IHD		26,547 (10.0)	25,307 (9.8)	482 (13.6)	621 (14.9)	137 (20.3)	<0.001
Incident AMI		2,356 (0.8)	2,207 (0.8)	49 (1.3)	82 (1.9)	18 (2.6)	<0.001
Incident angina pectoris		20,912 (7.8)	19,954 (7.7)	373 (10.5)	483 (11.6)	102 (15.1)	<0.001

Values are presented as mean±standard deviation, median (interquartile range), or number (%). PY, person-year; IHD, ischemic heart disease; AMI, acute myocardial infarction; BMI, body mass index; BP, blood pressure; AST, aspartate aminotransferase; ALT, alanine aminotransferase; GGT, γ-glutamyltransferase.

1 Negative: negative → negative, resolved: proteinuria ≥1+ → negative, incident: negative → proteinuria ≥1+, persistent: proteinuria ≥1+ → proteinuria ≥1+.

2 By analysis of variance test for continuous variables and chi-square test for categorical variables.

**Table 2.** Hazard ratios and 95% confidence intervals for incident ischemic heart disease according to changes in proteinuria

Variables		Person-years	Incidence cases	Incidence density (per 10,000 person-year)	Unadjusted	Multivariate adjusted^1^
Changes in proteinuria^2^						
	Negative	1,390,097.8	25,307	182.0	1.000 (reference)	1.000 (reference)
	Resolved	18,775.2	482	256.7	1.409 (1.287, 1.541)	1.211 (1.104, 1.329)
	Incident	21,551.3	621	288.1	1.580 (1.459, 1.711)	1.288 (1.184, 1.400)
	Persistent	3,326.0	137	411.9	2.258 (1.910, 2.670)	1.578 (1.324, 1.881)
p for trend					<0.001	<0.001

Values are presented as hazard ratio (95% confidence interval).

1 The multivariate model was adjusted for age, sex, body mass index, systolic blood pressure, fasting blood glucose, total cholesterol, γ-glutamyltransferase, smoking status, alcohol intake, physical activity, antiplatelet medications, and anticoagulant medications.

2 Negative: negative → negative, resolved: proteinuria ≥1+ → negative, incident: negative → proteinuria ≥1+, persistent: proteinuria ≥1+ → proteinuria ≥1+.

**Table 3.** Hazard ratios and 95% confidence intervals for incident acute myocardial infarction according to changes in proteinuria

Variables		Person-years	Incident cases	Incidence density (per 10,000 person-year)	Unadjusted	Multivariate adjusted^1^
Changes in proteinuria^2^						
	Negative	1,462,809.1	2,207	15.1	1.000 (reference)	1.000 (reference)
	Resolved	20,160.1	49	24.3	1.608 (1.211, 2.134)	1.401 (1.048, 1.872)
	Incident	23,223.1	82	35.3	2.336 (1.874, 2.913)	1.606 (1.268, 2.035)
	Persistent	3,691.4	18	48.8	3.225 (2.029, 5.127)	2.069 (1.281, 3.342)
p for trend					<0.001	<0.001

Values are presented as hazard ratio (95% confidence interval).

1 The multivariate model was adjusted for age, sex, body mass index, systolic blood pressure, fasting blood glucose, total cholesterol, γ-glutamyltransferase, smoking status, alcohol intake, physical activity, antiplatelet medications, and anticoagulant medications.

2 Negative: negative → negative, resolved: proteinuria ≥1+ → negative, incident: negative → proteinuria ≥1+, persistent: proteinuria ≥1+ → proteinuria ≥1+.

**Table 4.** Hazard ratios and 95% confidence intervals for incident angina pectoris according to changes in proteinuria

Variables		Person-years	Incident cases	Incidence density (per 10,000 person-year)	Unadjusted	Multivariate adjusted^1^
Changes in proteinuria^2^						
	Negative	1,407,427.2	19,954	141.8	1.000 (reference)	1.000 (reference)
	Resolved	19,119.9	373	195.1	1.375 (1.241, 1.523)	1.184 (1.065, 1.316)
	Incident	22,010.6	483	219.4	1.546 (1.412, 1.692)	1.275 (1.160, 1.401)
	Persistent	3,428.9	102	297.5	2.095 (1.725, 2.545)	1.554 (1.272, 1.899)
	p for trend				<0.001	<0.001

Values are presented as hazard ratio (95% confidence interval).

1 The multivariate model was adjusted for age, sex, body mass index, systolic blood pressure, fasting blood glucose, total cholesterol, γ-glutamyltransferase, smoking status, alcohol intake, physical activity, antiplatelet medications, and anticoagulant medications.

2 Negative: negative → negative, resolved: proteinuria ≥1+ → negative, incident: negative → proteinuria ≥1+, persistent: proteinuria ≥1+ → proteinuria ≥1+.

## SUPPLEMENTARY MATERIALS

Supplementary Material 1. Hazard ratios and 95% confidence intervals for incident ischemic heart disease according to changes in proteinuria after excluding the possibility of 1-year reverse causality (N=259,592)

Supplementary Material 2 Hazard ratios and 95% confidence intervals for incident acute myocardial infarction according to changes in proteinuria after excluding the possibility of 1-year reverse causality (N=264,798)

Supplementary Material 3 Hazard ratios and 95% confidence intervals for incident angina pectoris according to changes in proteinuria after excluding the possibility of 1-year reverse causality (N=261,011)
