# Supplementary material for: Changes in proteinuria and the associated risks of ischemic heart disease, acute myocardial infarction, and angina pectoris in Korean population
Source: Epidemiol Health. 2023 Sep 30;45:e2023088. doi: 10.4178/epih.e2023088 (PMC10867523; doi:10.4178/epih.e2023088)
Supplement: Supplementary Material 1. — Hazard ratios and 95% confidence intervals for incident ischemic heart disease according to changes in proteinuria after excluding the possibility of 1-year reverse causality (N=259,592) [file epih-45-e2023088-Supplementary-1.docx]

**Supplementary Materials 1.** Hazard ratios and 95% confidence intervals for incident ischemic heart disease according to changes in proteinuria after excluding the possibility of 1-year reverse causality (N=259,592)

|  | Person-year | Incidence  cases | Incidence density  (per 10,000 person-year) | Hazard ratios (95% Confidence Interval) | |
| --- | --- | --- | --- | --- | --- |
|  |  |  |  | Unadjusted | Multivariate adjusted |
| Changes in proteinuria |  |  |  |  |  |
| Negative | 1,387,650.7 | 19,931 | 143.6 | 1.00 (reference) | 1.00 (reference) |
| Improved | 18,730.3 | 378 | 201.8 | 1.408 (1.272-1.559) | 1.227 (1.105-1.362) |
| Incident | 21,491.7 | 492 | 228.9 | 1.604 (1.467-1.754) | 1.314 (1.196-1.443) |
| Persistent | 3,311.4 | 102 | 308.0 | 2.173 (1.789-2.640) | 1.572 (1.284-1.924) |
| *P* for trend |  |  |  | <0.001 | <0.001 |

Multivariate adjusted model was adjusted for age, sex, BMI, systolic BP, fasting blood glucose, total cholesterol, GGT, smoking status, alcohol intake, physical activity, anti-platelets medications and anti-coagulants medications.

Negative: negative → negative, Improved: proteinuria **≥** 1+ → negative, Incident: negative → proteinuria **≥** 1+, Persistent: proteinuria **≥** 1+ → proteinuria **≥** 1+
