# Supplementary material for: Changes in proteinuria and the associated risks of ischemic heart disease, acute myocardial infarction, and angina pectoris in Korean population
Source: Epidemiol Health. 2023 Sep 30;45:e2023088. doi: 10.4178/epih.e2023088 (PMC10867523; doi:10.4178/epih.e2023088)
Supplement: Supplementary Material 2 — Hazard ratios and 95% confidence intervals for incident acute myocardial infarction according to changes in proteinuria after excluding the possibility of 1-year reverse causality (N=264,798) [file epih-45-e2023088-Supplementary-2.docx]

**Supplementary Materials 2.** Hazard ratios and 95% confidence intervals for incident acute myocardial infarction according to changes in proteinuria after excluding the possibility of 1-year reverse causality (N=264,798)

|  | Person-year | Incidence  cases | Incidence density  (per 10,000 person-year) | Hazard ratios (95% Confidence Interval) | |
| --- | --- | --- | --- | --- | --- |
|  |  |  |  | Unadjusted | Multivariate adjusted |
| Changes in proteinuria |  |  |  |  |  |
| Negative | 1,462,614.2 | 1,797 | 12.3 | 1.00 (reference) | 1.00 (reference) |
| Improved | 20,157.3 | 40 | 19.8 | 1.610 (1.177-2.203) | 1.396 (1.011-1.927) |
| Incident | 23,217.2 | 69 | 29.7 | 2.422 (1.905-3.081) | 1.669 (1.289-2.162) |
| Persistent | 3,687.3 | 12 | 32.5 | 2.657 (1.506-4.687) | 1.852 (1.048-3.274) |
| *P* for trend |  |  |  | <0.001 | 0.005 |

Multivariate adjusted model was adjusted for age, sex, BMI, systolic BP, fasting blood glucose, total cholesterol, GGT, smoking status, alcohol intake, physical activity, anti-platelets medications and anti-coagulants medications.

Negative: negative → negative, Improved: proteinuria **≥** 1+ → negative, Incident: negative → proteinuria **≥** 1+, Persistent: proteinuria **≥** 1+ → proteinuria **≥** 1+
