# Supplementary material for: Changes in proteinuria and the associated risks of ischemic heart disease, acute myocardial infarction, and angina pectoris in Korean population
Source: Epidemiol Health. 2023 Sep 30;45:e2023088. doi: 10.4178/epih.e2023088 (PMC10867523; doi:10.4178/epih.e2023088)
Supplement: Supplementary Material 3 — Hazard ratios and 95% confidence intervals for incident angina pectoris according to changes in proteinuria after excluding the possibility of 1-year reverse causality (N=261,011) [file epih-45-e2023088-Supplementary-3.docx]

**Supplementary Materials 3.** Hazard ratios and 95% confidence intervals for incident angina pectoris according to changes in proteinuria after excluding the possibility of 1-year reverse causality (N=261,011)

|  | Person-year | Incidence  cases | Incidence density  (per 10,000 person-year) | Hazard ratios (95% Confidence Interval) | |
| --- | --- | --- | --- | --- | --- |
|  |  |  |  | Unadjusted | Multivariate adjusted |
| Changes in proteinuria |  |  |  |  |  |
| Negative | 1,405,586.3 | 15,924 | 113.3 | 1.00 (reference) | 1.00 (reference) |
| Improved | 19,089.3 | 299 | 156.6 | 1.384 (1.235-1.552) | 1.206 (1.072-1.356) |
| Incident | 21,966.1 | 392 | 178.5 | 1.584 (1.433-1.750) | 1.306 (1.175-1.451) |
| Persistent | 3,416.0 | 72 | 210.8 | 1.877 (1.489-2.366) | 1.427 (1.126-1.808) |
| *P* for trend |  |  |  | <0.001 | 0.001 |

Multivariate adjusted model was adjusted for age, sex, BMI, systolic BP, fasting blood glucose, total cholesterol, GGT, smoking status, alcohol intake, physical activity, anti-platelets medications and anti-coagulants medications.

Negative: negative → negative, Improved: proteinuria **≥** 1+ → negative, Incident: negative → proteinuria **≥** 1+, Persistent: proteinuria **≥** 1+ → proteinuria **≥** 1+
